# 5G connected and automated driving: use cases, technologies and trials in cross-border environments

**DOI:** 10.1186/s13638-021-01976-6

**Published:** 2021-04-16

**Authors:** Dirk Hetzer, Maciej Muehleisen, Apostolos Kousaridas, Sokratis Barmpounakis, Stefan Wendt, Kurt Eckert, Andreas Schimpe, Johan Löfhede, Jesus Alonso-Zarate

**Affiliations:** 1 Deutsche Telekom AG/T-Systems, Berlin, Germany; 2 Ericsson GmbH, Herzogenrath, Germany; 3 Huawei Technologies, Munich Research Center, Munich, Germany; 4 Department of Informatics and Telecommunications, National and Kapodistrian University of Athens, Athens, Greece; 5 Orange Labs, Châtillon, France; 6 Robert Bosch GmbH, Renningen, Germany; 7 Technical University of Munich, Munich, Germany; 8 Volvo Car Group, Gothenburg, Sweden; 9 i2CAT Foundation, Barcelona, Spain

**Keywords:** 5G, Connected and automated mobility, V2X, Automotive use cases, Cross-border, CCAM, Trials, QoS

## Abstract

Cooperative, connected and automated mobility (CCAM) across Europe requires harmonized solutions to support cross-border seamless operation. The possibility of providing CCAM services across European countries has an enormous innovative business potential. However, the seamless provision of connectivity and the uninterrupted delivery of real-time services pose technical challenges which 5G technologies aim to solve. The situation is particularly challenging given the multi-country, multi-operator, multi-telco-vendor, multi-car-manufacturer and cross-network-generation scenario of any cross-border scenario. Motivated by this, the 5GCroCo project, with a total budget of 17 million Euro and partially funded by the European Commission, aims at validating 5G technologies in the Metz-Merzig-Luxembourg cross-border 5G corridor considering the borders between France, Germany and Luxembourg. The activities of 5GCroCo are organized around three use cases: (1) Tele-operated Driving, (2) high-definition map generation and distribution for automated vehicles and (3) Anticipated Cooperative Collision Avoidance (ACCA). The results of the project help contribute to a true European transnational CCAM. This paper describes the overall objectives of the project, motivated by the discussed challenges of cross-border operation, the use cases along with their requirements, the technical 5G features that will be validated and provides a description of the planned trials within 5GCroCo together with some initial results.

## Introduction

Mobility is ongoing a profound transformation. Four are the main drivers for this paradigm change: (1) sharing economy, (2) electrification of vehicles, (3) driving automation and (4) connectivity.

In this paper, we focus on the potential of high automated driving and connectivity; in combination, they can enable the concept of Cooperative, Connected and Automated Mobility (CCAM). The realization of CCAM has the potential to bring safer mobility by reducing accidents, improve road traffic efficiency, reduce environmental impact of road traffic and foster new ways of creating revenue and reducing both capital and operational costs of mobility stakeholders.

Indeed, the exchange of information among vehicles, pedestrians and road infrastructure—facilitated by the so-called V2X (*vehicle-to-anything*) communications—allows extending the vision and detection range of on-board sensors even when visual line-of-sight (LoS) is not available due to obstacles, geometry or weather conditions. This extension of sensing capabilities leads to the possibility of making smarter decisions and allowing vehicles to actuate in a highly automated manner, thus reducing human error and boosting safety, driving efficiency and passenger comfort.

However, the realization of CCAM poses various challenges on different fronts, being technology readiness one of them (but not the only one). In order to solve them, all involved stakeholders must cooperate together to define solutions and overcome existing hurdles. For this reason, the telco and automotive worlds must team up to design and deploy solutions which can satisfy the requirements of CCAM, while at the same time respecting regulation and the interests and needs of stakeholders.

Motivated by this, different standardization organizations (3GPP [1], ISO [2], ETSI ITS [3]), associations (e.g. 5GAA [4]) and research projects around the world (e.g. 5G-PPP [5]) are working on making possible all elements needed for CCAM.

One of the primary challenges consists in ensuring that communication networks can satisfy the communication requirements of CCAM services. These include the need for real-time response and ultra-high reliability. On top of that, when pan-European transportation paths are considered—the so-called corridors—there is a need to ensure that these requirements can also be satisfied when vehicles drive across national borders. In such context, service continuity must be guaranteed, and the quality of service (QoS) must either not be degraded below what the service can accept when crossing borders or quality degradation must be anticipated—predicted—in order to take corrective actions, e.g. disabling a safety–critical service which relies on connectivity. The situation is particularly challenging considering that changing country may also imply changing the serving mobile network operator (MNO), the actual telco equipment provider, the enforced road traffic regulations and, in addition, services may run for all car manufacturers.

Motivated by this challenging situation, 5GCroCo [14] is an Innovation Action, partially funded by the European Commission, which aims at contributing to the development and trials of 5G technologies for cross-border CCAM in Europe. The time frame of the activities of 5GCroCo comprises from November 2018 to the end of 2021.

The overall aim of 5GCroCo is to contribute to the definition of a successful path towards the deployment of CCAM infrastructures and services in cross-border scenarios and to reduce the uncertainties of real 5G cross-border deployments. 5GCroCo will trial 5G technologies in the Metz-Merzig-Luxembourg cross-border corridor, which involves the borders between France, Germany and Luxembourg. The objective is to validate advanced 5G features in the cross-border context, such as 5G new radio, mobile edge computing/cloud (MEC), predictive quality of service, software-defined networking (SDN), network slicing and improved positioning systems, all combined together to guarantee that innovative use cases for CCAM can be enabled. In addition, 5GCroCo aims at defining new business models that can be built on top of this unprecedented connectivity and service provisioning capacity, also ensuring that relevant standardization bodies from the two involved industries are impacted. 5GCroCo validation is focused on three use cases: (1) tele-operated driving (ToD), (2) high definition (HD) map generation and distribution for automated vehicles (in short, HD mapping) and (3) anticipated cooperative collision avoidance (ACCA). Moreover, it will also provide general recommendations for other CCAM use cases.

The remainder of the paper is organized as follows: Sects. 2 and 3 provide a project overview of 5GCroCo along with the experimental methodology. The use cases that are tested in 5GCroCo are presented in Sect. 4. Section 5 describes the key technologies as enablers for CCAM that will be validated. Section 6 presents the trials that will be conducted at both small and large scales, along with an overview of Key Performance Indicators (KPIs) that are considered for the different trials and respective measurement tools. Business models and cost/benefit analysis are discussed in Sect. 7. Some first results are presented in Sect. 8. Section 9 concludes the paper.

## 5GCroCo project overview

5GCroCo is an innovation action partially funded by the European Commission (EC) under the umbrella of the 5G Public Private Partnership (5G-PPP). The main goal of this 5GPPP is to coordinate and lead the development of 5G technologies in Europe. 5GCroCo is devoted to conduct large-scale trials of 5G technologies for CCAM in the European 5G cross-border corridor connecting the cities of Metz (in France), Merzig (in Germany) and Luxembourg. In addition to the large-scale trials in the 5G corridor, 5GCroCo has also conducted small-scale tests and pilots in Barcelona, Montlhéry-UTAC, Munich, AstaZero and the German A9 Motorway 5G-ConnectedMobility testbed.

5GCroCo has a total budget of close to 17 million euros and counts with a contribution from the EC close to 13 million euro, with a planned duration of 3 years, running from beginning of November 2018 until end of October 2021. The project is coordinated by the Centre Tecnològic de Telecomunicacions de Catalunya (CTTC, in Castelldefels, Barcelona, Spain), and gathers 24 partners from 7 European countries comprising key organizations in the intersection of both the telecom and the automotive worlds. 5GCroCo coordinates the contributions from leading car manufacturers, tier-1 suppliers, road authorities, mobile network operators, telecom vendors, small and medium enterprises (SME) and academia.

The trials of 5GCroCo will focus on three use cases related to CCAM services: ToD, HD mapping and ACCA. The next section describes them in further detail.

## Experimental methodology

The activities of 5GCroCo are organized in two iterations, both ending with a trial phase to collect results in the test and trial sites and analyse them. Baseline for the trials is the architecture including the 5G technologies described in Sect. 5. These architectures are generic and reflect how the project envisions real commercial deployments of CCAM. Trials built based on these architectures try to reflect the real world as close as possible, but limitations apply, as true for every trial. An essential part of the result analysis is, therefore, a discussion if and how results would differ in a realistic commercial deployment by means of extrapolation.

Key Performance Indicators (KPIs) described in Sect. 5.4 are mapped to end-to-end network performance parameters. Further parameters, e.g. per link latencies, are measured to understand main factors influencing end-to-end performance and allow for improvements to be designed and then trialed in the second trial round.

## 5GCroCo use cases

5GCroCo aims at validating three CCAM use cases in cross-border situations. The use cases have been selected to ensure that they allow testing the need for high-performance 5G features and the need for cross-border operation. As it will be explained later, the three use cases are complementary of each other, focusing on different 5G features.

While the actual trials and validations in 5GCroCo focus on these particular use cases with envisioned high potential market opportunities, the activities of 5GCroCo aim at deriving recommendations and insights which can be valid for a wider set of CCAM use cases.

KPIs have been defined to allow conducting the trials and defining the measurements to be done.

### Tele-operated driving (ToD)

**Fig. 1:**
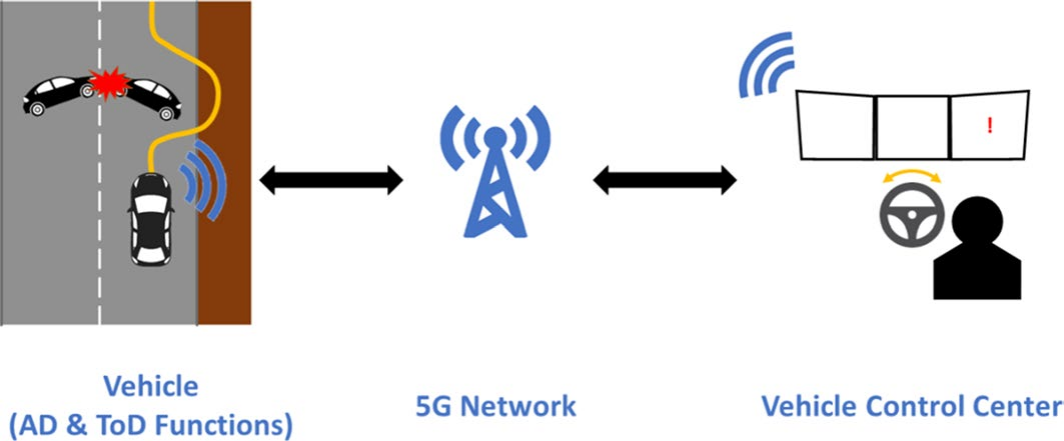
Tele-operated driving (ToD)

ToD is defined as the remote control or support of automated vehicles by a human in a vehicle control center over a mobile radio network, as illustrated in Fig. 1. ToD in the context of automated driving can be deployed in different traffic situations, such as:

- *Non-responding driver:* even though Level 4 automated driving vehicles are not able to handle every situation, the driver is not either required to be always ready to regain control as with Level 3 autonomous driving. In the case that the driver does not respond to the request of taking over control, an operator in a vehicle control center can take over control.
- *Handling in special areas:* a vehicle could be remotely operated in special situations or areas. For example, at freight centers, a Level 3 truck could be remotely handled by a tele-operator in order to allow the driver to take his recreation time during the period of loading or unloading freight.
- *Undefined traffic situations:* in the event of a highly automated driving-enabled vehicle (Level 4) not capable to handle a certain traffic situation, ToD can remotely involve a human operator to solve the situation. This could happen through providing additional information to the vehicle, enabling it to correctly perceive its situation. It can also include temporarily taking over control to resolve the situation or proposition of a new route.

With the 4G/LTE mobile network standard, it became conceivable that tele-operation of road vehicles could be a feasible undertaking. Startups, e.g. Phantom Auto [15], emerged. Also, larger companies, such as Ericsson in cooperation with Scania [16], or Huawei in cooperation with SAIC Motors [17], are working on tele-operation. However, until today, there do not yet exist business models for ToD. It demands a high reliability of the communications network that may not be achieved with the current 4G/LTE mobile network standard. However, with 5G technologies promising to drastically reduce network latency and increase reliability, this is subject to change. Furthermore, seamless service along the route, where the vehicle is tele-operated, can be enabled with methods to predict the foreseen Quality of Service.

Within 5GCroCo, a number of variants to perform the ToD use case have been defined. These variants are further referred to as user stories, and they include:

- User stories applying **direct control** of the remotely driven vehicle: in these user stories, the remote operator is directly maneuvering the remote vehicle by accessing the actuators in the vehicle, e.g. through usage of remote steering wheels or pedals or other manipulation devices.
- User stories applying **indirect control** of the remotely driven vehicle: in these user stories, the remote vehicle keeps control of the direct maneuvering itself but gets additional information or allowances from the remote operator. For instance, this could be generating and sending a new driving path that helps the vehicle to overcome a blocked road situation.

Several challenges arise to realize ToD. This use case imposes high bandwidth demands for uplink video streaming and the need to provide the capacity to control several vehicles in an area. An extra challenge arises from the fact that there is a high risk of correlation of requests for remote drivers to take over control. This is caused by previously described situations for ToD, making it likely that if one vehicle requests the service, other vehicles in the same area will request it as well. Furthermore, involved network infrastructure, as well as backend servers running the application services required for this use case, will likely belong to different domains, e.g. different MNOs. If communication or service orchestration is required across those domains, QoS requirements must still be met end-to-end. This also applies when country borders are crossed. The network should be designed in a way to provide the required QoS with very high reliability. Predictive QoS is required to issue warnings towards the people in the vehicle and the remote operator in the vehicle control center, when potential QoS degradation that may occur in the near future will no longer be capable of addressing the tele-operated service requirements in terms of data rate, latency or availability. Possible consequences depend on previously described situations and automated driving levels, but may include reducing speed, changing the route towards one where the network can support the service, or safely stopping the car. On top of QoS prediction, which is an important base requirement for ToD, the safe remote control of a vehicle requires an overall functional safety concept. This concept development needs to consider the in-vehicle architecture, the 5G communication network architecture with its advanced features and the backend architecture.

The KPIs of ToD are low end-to-end latency, high reliability and high uplink data rates. For uplink, the major requirement is to transmit reliable and in-time video information. For this, it is key that the video has a reasonable resolution, only minor artifacts and a low latency. The transmission in uplink therefore needs to provide a combination of reliable high data rates and reliable low latencies as well as a low number of packet errors. In downlink, the commands being sent from the operator to the vehicle need to be in-time and with no end-to-end errors leading to a combination of zero undetected errors while keeping the latency at a reasonable low value. Preliminary results of the ToD system and the KPI collection are presented in Sect. 8.

### High definition (HD) map generation and distribution for automated driving

**Fig. 2:**
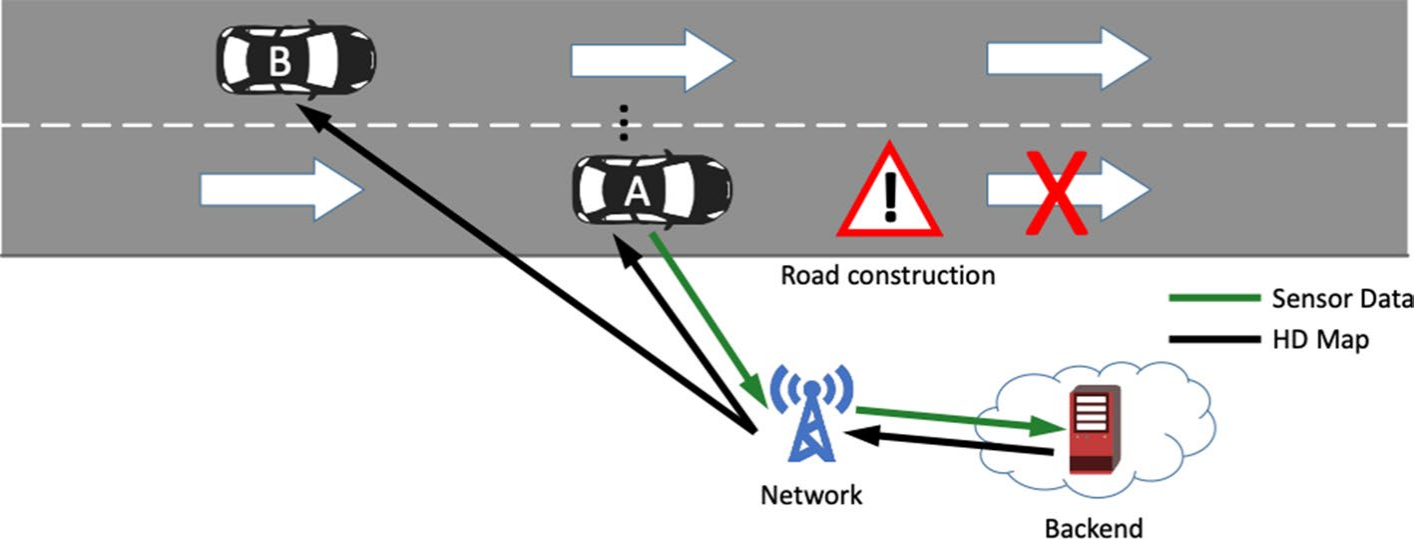
High definition mapping (HD mapping)

Intelligent and dynamic HD maps, exemplified in Fig. 2, provide highly accurate position of dynamic and static objects which enable tactical and operational planning by an autonomously or semi-autonomously driven vehicle. Such maps could be constructed by smartly fusing all the available data from different sources at and along the roads, e.g. the sensor data shared by the vehicles, data shared by the road infrastructure or by map content providers, among others.

5GCroCo tests 5G technology for exchanging the data required to generate the HD maps between the vehicles, data servers and map providers. The focus is on using sensor information, harvested in real time from cars, to update a cloud-based HD map. The sensor information can, e.g. be used to correct a static map feature that has changed, for example when a lane line has been repainted or a barrier has been broken or added. It could also be used for populating the map with more dynamic information, e.g. road works. This type of information will be of high importance for an autonomous or semi-autonomous vehicle, for example, by warning the driver that he or she needs to take over if the car anticipates an event that is outside of its operational design domain.

The main KPI for HD mapping is the on-time availability of up-to-date HD map information about an area right before entering that area. This corresponds to minimum throughput requirements for the network. Again, here the combination of data rate, data quality, seamless coverage and latency is what matters. Information must not be too old and, therefore, cannot be uploaded too much in advance. On the other hand, information exchange needs to start early enough to be finalized with no remaining errors before entering the area of interest.

### Anticipated cooperative collision avoidance (ACCA)

At high speed, a typical stand-alone sensing system (e.g. radars, cameras, lidars) will not have sufficient and safe means to detect and localize dangerous events on the road in all situations and with sufficient level of anticipation. In such situations, too late detection of a dangerous event will trigger a hard braking and, possibly, a collision, depending on friction conditions. ACCA techniques can solve this kind of situations by allowing cooperation among vehicles.

In particular, 5GCroCo defines, tests and trials cooperative solutions to anticipate the detection and localization of such dangerous events and to facilitate smoother and more homogeneous vehicle reaction. This is called ACCA and can be useful in several situations, such as:

- Temporarily static events like traffic jams.
- High deceleration, emergency braking or unexpected maneuver of vehicles ahead (with or without visibility for the ego vehicle).
- Cut-in anticipation, e.g. when a vehicle suddenly comes in from another lane.

The cooperative vehicles (or the roadside infrastructure, for example) will upload a set of information such as status (e.g. position, speed, acceleration), detected events and some sensor data (camera/radar streams, or any other information based on a standardized methodology, e.g. Cooperative Perception Messages) towards specific servers. These data will be used by local, MEC-hosted servers, to create an off-board dynamic map which handles and consolidates all collected information based on a known road topology. The off-board distributed service is used to:

- Aggregate and consolidate data received from vehicles with data coming from, e.g. road operators.
- Manage independently (on a per-vehicle or on a per-geographical area) dynamic information, especially in areas with traffic congestion.
- Extrapolate from the distributed dynamic map the relevant content for a specific user.
- Ensure the hand-over towards neighbouring MEC hosts.
- Ensure roaming with seamless service continuity between different countries and MNOs.

**Fig. 3:**
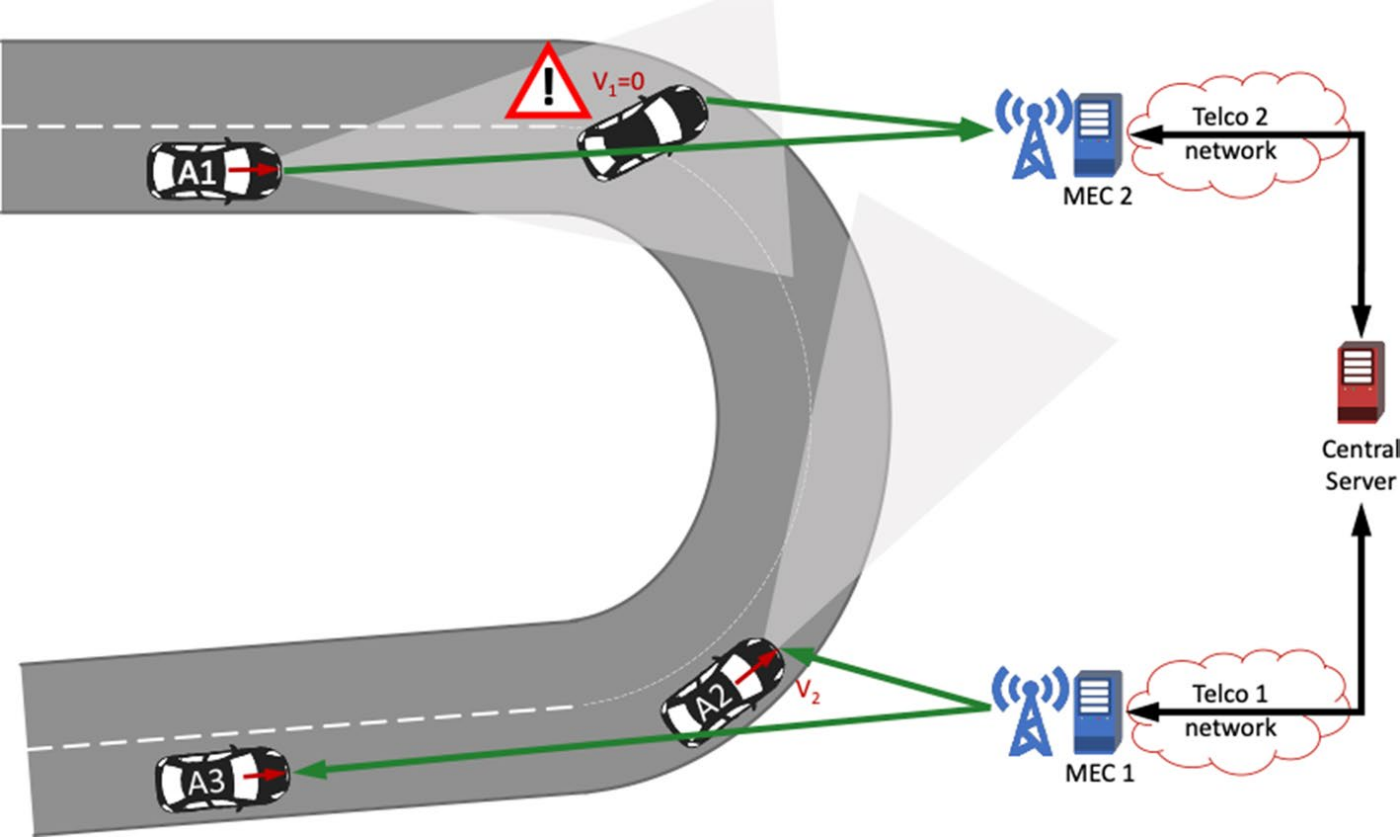
Anticipated cooperative collision avoidance

The main KPIs for this use case are the need for updated information and very high reliability. This corresponds to low delay and very low packet loss ratio for the communications network. It is of utmost importance that the information about a seriously dangerous situation is communicated to all other vehicles in the area of relevance early enough and with a data “age” that is small enough so that the endangered vehicles can perform reasonable maneuvers in time. This means, therefore, that the combination of reasonable low latency, reasonable communication quality and seamless coverage of at least the road networks are key for the successful operation of the ACCA service (Fig. 3).

## 5G technologies in 5GCroCo

The use cases defined in the previous section are used as carriers to test and trial 5G functionalities in 5GCroCo. Indeed, 5GCroCo has identified a set of key 5G technologies which will become key enablers for CCAM. Individually, most of them have all been thoroughly evaluated in previous and ongoing research and innovation projects. Some of them are even commercially deployed already. The motivation of 5GCroCo is to evolve them to also fulfill their purpose and role in overall QoS fulfillment in cross-border, cross-MNO, cross-telco-vendor and cross-OEM deployments. A detailed description of the 5GCroCo network architecture can be found in [20]. Uninterrupted service continuity is a goal in this context. The key identified technologies are:

- MEC
- Predictive QoS
- E2E QoS with Network Slicing
- Mobile Network-Supported Precise Positioning
- Security.

The V2X services that are studied and trialed in 5GCroCo for the selected use cases have unique characteristics which make the use of these technologies particularly interesting. These are discussed as follows.

First, a limited area of interest: in the considered use cases, information is generally only needed close to the source where it was generated. This is true for many, but not all applications. It particularly applies to the use cases of HD mapping and ACCA. Direct communication omitting the cellular network and MEC-enabled cellular networks must be, therefore, part of the V2X architecture.

The second unique property is the multi-OEM and multi-MNO challenge. This one is tightly coupled with the first one. For typical mobile radio networks providing services like voice and data communication, it does not matter that peering points between MNOs, vehicular clouds and public data networks are located far from the “edge”. However, in a MEC-enabled V2X architecture, this problem must be solved, and the solution cannot be to have just one MEC provider.

The third unique property is the role of the road authority as another source and sink of information. This comes along with often closed, sometimes even proprietary, information technology (IT) systems needing integration in a MEC-enabled distributed computing V2X network architecture. A particular challenge arising from this is that crossing national, in some cases also regional, borders results in a new road authority with own IT infrastructure becoming responsible.

With these technologies, 5GCroCo addresses the gap of existing cellular V2X technologies (e.g. LTE Release 16) by enhancing a number of KPIs in the 5G network, such as latency, reliability, packet error rate, etc., even under cross-country, cross-MNO, cross-OEM and cross-telco-vendor operations.

Recent 3GPP studies on 5G New Radio (NR) [6] enabled the evolution of 4G Evolved Packet System (EPS) architecture towards non-standalone 5G NR, i.e. via gNB-based user plane communication, supported by the eNB functionality for the control plane. Towards the same direction, 3GPP identified architectural enhancements for the control plane and user plane separation of EPC nodes [7].

A number of architecture enhancements for V2X services -from the architectural, service requirements and application layer support aspects, respectively [8]-[10], have been already identified by 3GPP in Release 16 as well. In particular, the means to support QoS prediction, via enhancements of 5G System (e.g. via N33-interface) are already being studied by the 3GPP Service Architecture (SA) Working Group (WG) (5GS) [11].

The goal is to enable 5G communication systems to provide analytics information regarding potential QoS change upon request from a V2X Application Server, which also provides the network with information about location information, time window indicating the time period to which the information in the potential QoS change notification applies and threshold(s), indicating level(s) which, if crossed, trigger the notification that the potential QoS change (improvement or worsening) can happen.

The MEC paradigm is also considered a potential solution towards achieving ultra-high reliability and ultra-low latency requirements in CCAM services. Towards exploiting the MEC enabler, ETSI described several architectural enhancements [12], such as the “Distributed S-/P-GW” deployment option, where extra serving and packet data network (PDN) gateways (S-GWs and P-GWs respectively) are used to realize access to the MEC host. Different aspects of MEC management and application lifecycle, rules and requirements have been also studied by ETSI [13], which apply directly for V2X MEC deployments. End-to-end network services that are built through network service descriptors (NSDs) with a set of different virtual network functions (VNFs) and MEC application descriptors (MEC AppDs) [13] can thus be exploited.

**Fig. 4:**
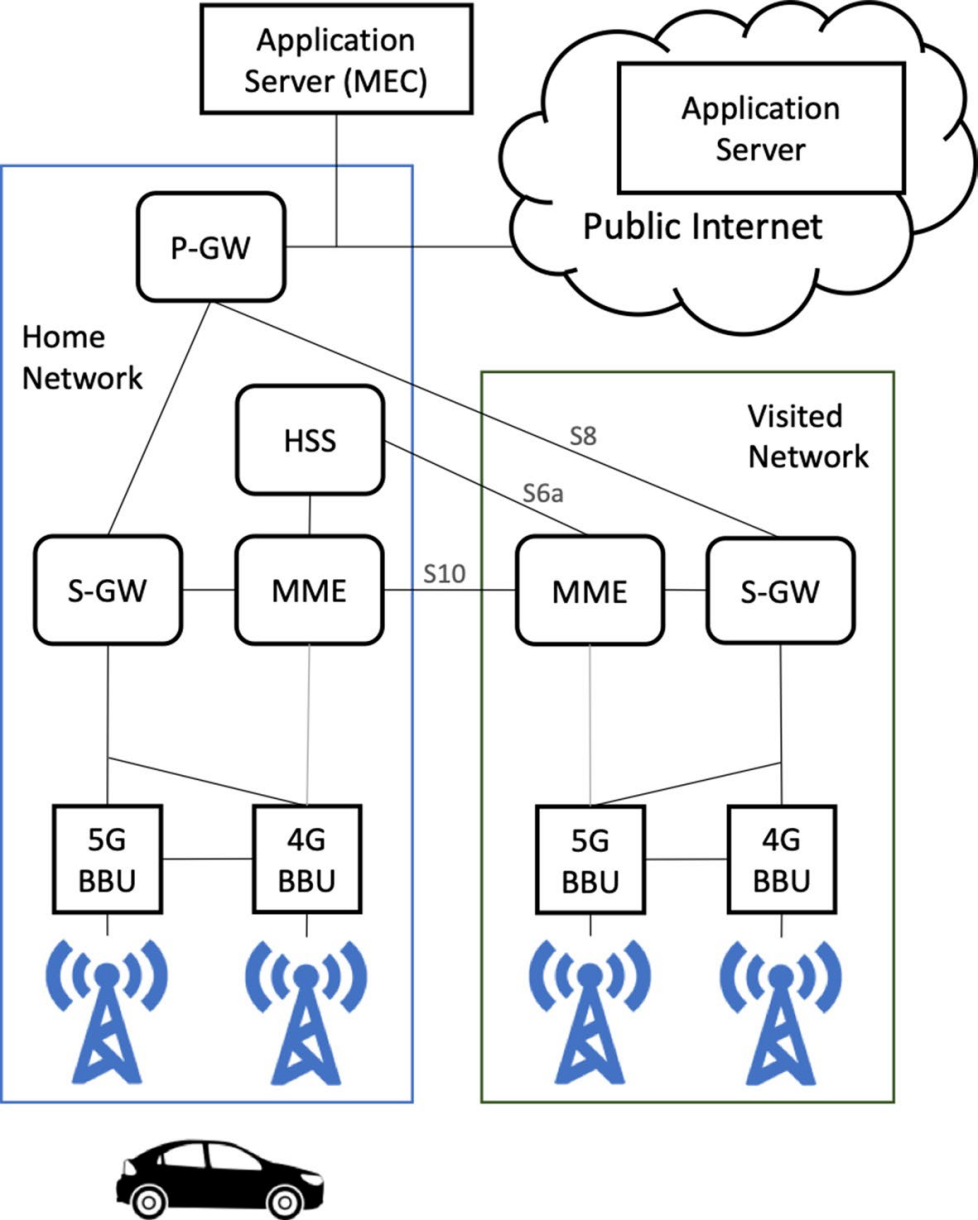
High level architecture deployed for 5GCroCo trials

An overview of parts of the architecture that is used at the 5GCroCo trials is provided in [20]. Figure 4 focusses on the multi-MNO aspects and therefore shows only one MEC-host, as currently done for trials with Home Routed Roaming. The 5GCroCo architecture [20] and final trials consider multiple MEC-hosts within the same and across different MNOs and studies switching between these hosts and corresponding P-GWs (and user plane functions (UPFs) for standalone 5G NEW RADio) as the vehicle moves.

In the following sub-sections, we further elaborate on the challenges to be solved by each of these technologies.

### MEC—mobile edge cloud/computing

MEC is often associated with computational resources collocated with one or more base stations. The MEC definition used for the 5GCroCo project is not strictly bound to geographic location of the MEC, but to the fact that applications running on such MEC instance experience very low delay to and from the base stations the MEC is related to. A local MEC instance serving a limited geographic area in combination with vehicle mobility results in frequent change of the MEC instance handling the particular vehicle (i.e. MEC handover). For this and other MEC-related challenges, 5GCroCo will define generic MEC architectures and best practices to deploy the micro-services required for the services forming the three project use cases and further ones typically encountered in V2X context. The architecture document [20] provides examples for the HD Mapping and ACCA use case listing events and triggers that are used to switch P-GWs/UPFs within and across MNOs as a vehicle moves between different cells. Service continuity aspects are described from application and network point of view. The goal is to reach an optimal trade-off between network performance and both deployment complexity and cost.

### Predictive quality of service (pQoS)

Safety of life can often be traded for availability or degraded performance. Brought into V2X context, it can for example mean increasing inter-vehicle distance and, therefore, reducing traffic throughput or even stopping the vehicle. For that, the state of the communication system must be monitored, failure must be detected and the application/service must be informed. Previous research activities (e.g. [6]) have shown that instantaneous information of network failure is often not enough to guarantee functional safety. The vehicle might require time to enter a safe state and might need to rely on communication while doing this, e.g. to inform surrounding cars or query application services about the best possibility to reach a safe state in the current situation.

5CroCo ambition regarding predictive QoS is to broaden the scope from pure Radio Access Network (RAN) to end-to-end evaluation. Redundancy, as key for reliable communication, will be evaluated for RAN, core and backend, with particular focus on how MEC can ensure redundancy, failover and fast recovery mechanisms. 5GCroCo will extend the network with very reliable prediction of the QoS levels for different types of CCAM services that will be evaluated in the context of the project use cases. The QoS prediction enables in-advance information of the expected network quality for a given location and time window, for a specific subscriber or cluster of subscribers in the network.

Within the project, 5GCroCo determines the performance and applicability of existing predictive QoS algorithms by evaluating them in realistic scenarios and evolve underlying interfaces and architectures to enable cross-border and cross-MNO predictive QoS.

### E2E QoS with network slicing

Network Slicing is a technique which opens a wide set of options for MNOs to instantiate one or more virtual networks. While different standards (e.g. 3GPP Rel. 15, ETSI-MEC) and data center technologies pave a way to a convenient and interoperable flexible and controlled instantiation of multiple logical networks on top of a shared infrastructure, it is naturally not standardized how many and which kind of slices should be deployed and when.

5GCroCo addresses this question by considering the entire solution space, i.e. starting with an assumption of no dedicated V2X slice at all (very unlikely) and going to a more fine-grained differentiation resulting in more than one dedicated slices. 5GCroCo is evaluating the applicability of Slice Templates defined by GSMA [21].

### Mobile network supported precise localization

The advanced Intelligent Transportation System (ITS) services for cooperative, connected and automated cars developed in 5GCroCo require delivering cm-level accurate positioning to moving vehicles. This accuracy cannot be supported today with GNSS (Global Navigation Satellite Systems), especially in urban environments (e.g. due to urban canyons). 5GCroCo will design and validate innovations to deliver cm-level positioning supported by the 5G network.

5GCroCo will approach the positioning problem from a holistic perspective, considering approaches dependent and independent from the Radio Access Technology (RAT), and also hybrid approaches. As RAT-dependent approaches, 5GCroCo will study physical layer extensions to 5G NR (new radio) that allow a User Equipment (UE) to obtain the required accurate position.

### Security

It is a key goal of 5GCroCo to guarantee that state-of-the-art security standards are met throughout all areas of the involved IT infrastructure. The overall architecture, communication protocols, software and hardware should have appropriate measures implemented that ensure the maintenance of the security goals, including confidentiality, integrity, availability and authenticity. Strict adherence regarding data privacy in accordance with the General Data Protection Regulation (GDPR) is also an integral part.

Along the different stages of the project, security aspects including privacy, confidentiality, integrity, availability and authenticity have been [20] and will be further evaluated. Special focus will be put onto the first one, privacy, since here we expect most V2X (vehicle to anything)-specific challenges. Existing security mechanisms will be evaluated, and gaps will be identified. Solutions to close them will be defined, preferably relying on standard Internet or C-ITS-specific mechanisms defined in IETF, ETSI-ITS and other Standard Development Organizations.

## 5GCroCo test sites

5GCroCo large-scale trials will be conducted in 2021, while the majority of small-scale trials have been conducted in 2020, to validate the 5G technologies described in Sect. 4 for the three use cases described in Sect. 3.

### Large-scale cross-border corridor

5GCroCo will trial all the use cases at the European cross-border corridor which connects cities in France, Germany and Luxembourg, and is part of the pan-European network of 5G corridors facilitated through several regional agreements (see Fig. 4). These agreements allow Europe to count with hundreds of kilometers of motorways where tests can be conducted up to the stage where a car can drive autonomously with a driver present (Automated Driving Level 3 [18]). These corridors count with the support of the European Commission as part of its 5G Action Plan, which aims at ensuring commercial deployment of 5G technologies by the end of this decade [19].

5GCroCo will evaluate substantial parts of the architecture developed in the project according to the technologies presented in Sect. 4, which are deployment and configuration this. These large trials are planned to happen in two rounds. The first round is planned in Q2 2021. The final trail round is planned in Q4 2021.

### Small-scale tests and pilots

As rolling out such trials is a very complex task, a good step-by-step preparation has been adopted. 5GCroCo has several trial sites called “small-scale” trial sites. Indeed, these sites aim at preparing all use cases and user stories as a first step to check that everything is validated and working before continuing at large scale. This allows also dealing with all potential problems and issues we could be facing and allows to collect best practice. 5GCroCo has small-scale site in Germany, France, Spain and Sweden. Almost all planned small-scale tests and trials have been conducted in 2020.

These pilots have been deployed in a test track in Montlhéry-UTAC (South of Paris, France), two in Germany (in a section of Motorway A9 5G-ConnectedMobility test site and a test-site in the city center of Munich), one in the city of Barcelona (Spain) where a cross-border city setting will be emulated and one in Sandhult near Göteborg (Sweden) on the AstaZero test track, where also virtual border-crossing is implemented. These pilots allow testing 5G functionalities locally (often geographically close to the different involved partners), and possibly in restricted closed areas, so that the complexity of doing the trials in the large-scale corridor can be managed. In addition, these small-scale tests allow the fine-tuning the 5G capabilities for the large-scale trials, thus reducing the uncertainties associated to their deployment and trial.

### Trial timeline

A summary of the 5GCroCo timeline is provided in Table 1, indicating the different activities of the small-scale tests and the large-scale trials. The installation and verification of the 4G/5G networks at the different small-scale sites have been already finished in 2020. The use case-specific installations per small-scale site and the associated validation tests have been conducted mainly in Q2 and Q3 2020.

**Table 1:** 5GCroCo tests and trials timeline

	Small-scale	Large-scale
*2020*		
Q1	5G network setup	
Q2	UC setup	5G network setup
Q3	Tests	UC setup
Q4	Trials	Tests and trials
*2021*		
Q1	Preparation for large-scale tests and trials	UC/5G issue solving and trials
Q2		Finish trials—1st round, setup and conduct trials—2nd round
Q3		
Q4		

### Key performance indicators (KPIs)

5GCroCo monitors and evaluates the performance of the proposed 5G innovations in the trials based on the measurement of KPIs. KPIs related to telco operations will be considered. Those KPIs will be evaluated during the execution of all 5GCroCo trials, in a horizontal manner and will focus on the primary network infrastructure operations such as end-to-end connectivity, service range, mobility and resource management aspects, security, etc.; examples of such KPIs will be reliability, maximum speed supported by user equipment, connection establishment-related delay, etc. In addition to the horizontal network operation-related KPIs, each one of the trials will focus on a specific set of KPIs, tailored to the requirements and scenarios of the trial. Those use case-specific KPIs are formed from a service- or application-oriented aspect and will be measured via a diverse set of tools. The following KPIs have been identified:

- KPI #1: Maneuverings range
- KPI #2: Service range
- KPI #3: Information exchanged and estimated payload
- KPI #4: Application-level latency
- KPI #5: Application-level reliability
- KPI #6: Age of information
- KPI #7: Positioning accuracy
- KPI #8: Deviation from desired path
- KPI #9: Service provisioning time

Table 2 illustrates at which test and trial sites which KPIs are measured.

**Table 2:** KPIs to be evaluated per test and trial site

5G solution		Luxembourg–France–Germany cross-border	Montlhéry	Motorway A9	Munich	AstaZero	Barcelona
Use case	KPI						
ToD	1	X			X		
	2	X			X		
	3	X			X		
	4	X			X		
	5	X			X		
	6	X			X		
	7	X					
	8	X					
HD mapping	5	X		X		X	
	6	X				X	
ACCA	4	X	X				X
	5	X	X				X
	9						X

## Business innovations in new ecosystems for automated driving

In addition to the 5G trials for CCAM, the study and definition of new business models and cost/benefit analysis is a fundamental part of 5GCroCo to understand the business possibilities that emerge from CCAM services which can operate across borders. The possibility of having advanced 5G functions operating in a cross-border, cross-telco-vendor, cross-car-OEM, cross-MNO fashion generates a new arena for innovation. 5GCroCo will analyse the cost/benefit relationship of deploying 5G in such a complex scenario and develop tools which can allow for the definition of valid business models. This process will be done in parallel with the deployment of the trials, learning from the experience acquired, understanding the needs of all stakeholders and reducing the uncertainties of deploying a 5G infrastructure to offer unprecedented 5G-enabled services for CCAM.

## Results and discussion

In this section, first results of the ToD and the ACCA use cases are presented. It should be noted that due to the sanitary crisis COVID-19, the 5GCroCo tests and trials face delays. Due to the delay in trial realization, first results were available in January 2021. For the ToD use case, first validation tests of the system and KPI collection in a commercial 4G/LTE network were performed, in which results are presented in the following. For ACCA, trial results from the large-scale test and trial site are provided. Further results for these two use cases and for the HD Mapping use case can be found in [22].

### Tele-operated driving

In the following, first results of ToD use case tests, validating the system and the KPI collection, are presented. The tests were performed in a commercial 4G/LTE Network.

**Fig. 5:**
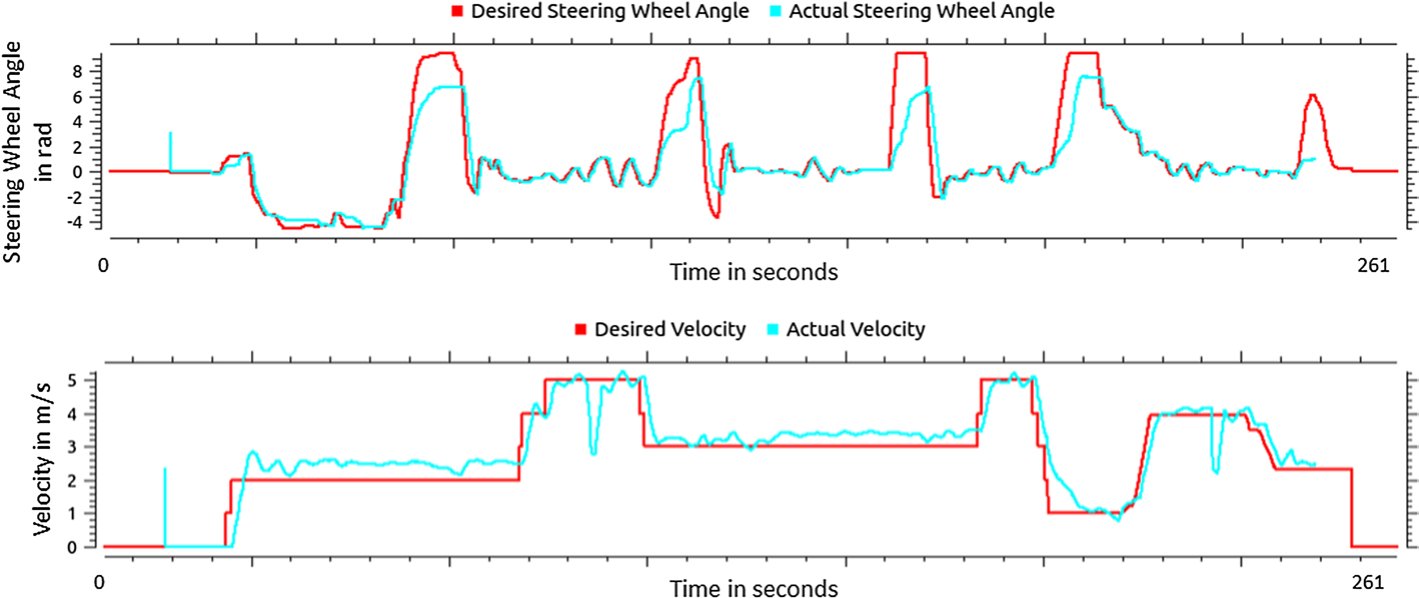
Tracking performance of the steering wheel angle and the velocity

In Fig. 5, the tracking performance of the steering wheel angle (top) and the velocity (bottom) during a ToD session are plotted over time. In red, the values requested by the operator are shown. The actual value of the respective quantity, as fed back by the vehicle, is shown in cyan. It is noticeable that the achieved steering wheel angle in the smaller angle ranges accurately follows the desired steering wheel angle. Remote-controlled steering of the vehicle is therefore possible with the necessary accuracy. Exceptions are fast and large changes of the desired steering angle, where it is not possible to follow them completely. In particular, the fully required steering wheel angle is not achieved.

Therefore, especially in situations in which comparable inputs arrive at the vehicle will lead to greater deviations between the intended and driven path. The reason for this is probably the limited steering force of the automated steering system, which is done for safety reasons. This could be counteracted, for example, by limiting the maximum steering angle in the VCoC. Similarly, the velocity tracking in the lower range (smaller 4 m/s) shows overshooting, and minor inaccuracies due to minor situation-dependent fluctuations (pavement, tire pressure, etc.). However, the tracking is deemed sufficiently accurate to successfully perform the task of ToD.

**Fig. 6:**
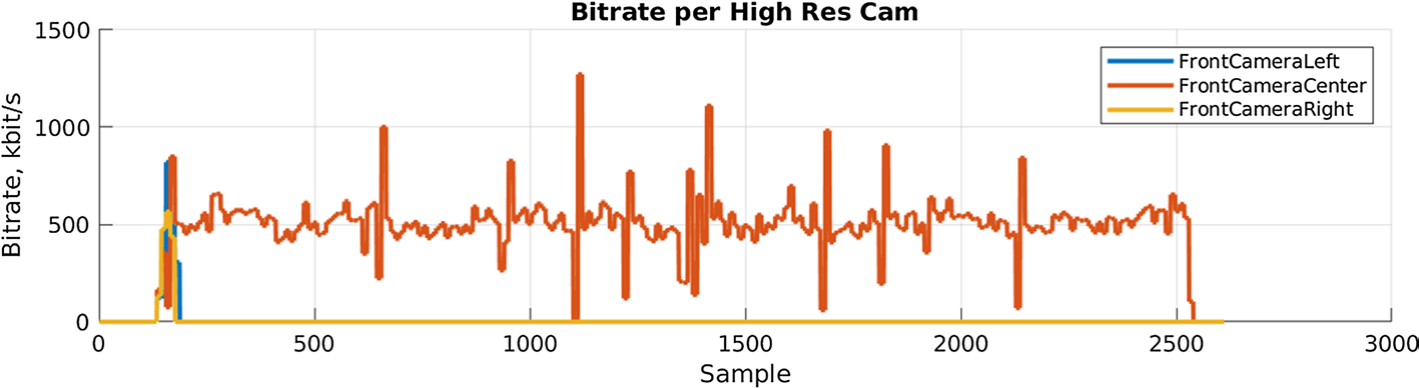
Bitrates of the three front-facing cameras on the vehicle measured in the VCoC

The camera bitrates of the three front-facing cameras on the vehicle during a ToD session are shown in Fig. 6, each color representing one camera. At first, multiple cameras were supposed to be used. However, insufficient network coverage led to the decision to only transmit a single video stream from the front-center camera at a bitrate of 500 kbit/s.

**Fig. 7:**
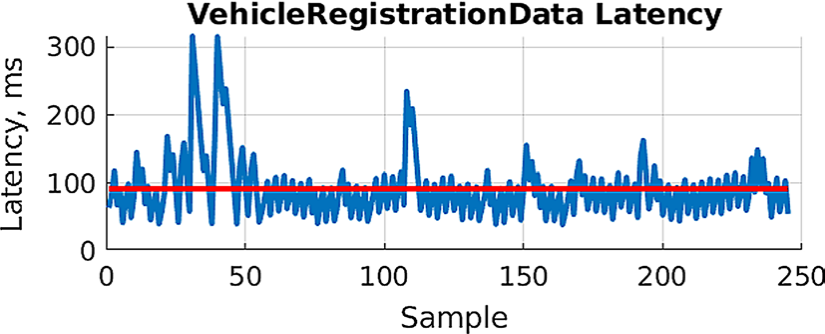
Application-level latency of the vehicle registration signal measured in the VCoC

The figure shows that all three cameras send data in the first few samples. For the front-center camera, a great variance in the data rate due to the unstable mobile network performance can be observed. This is also noticeable in Fig. 7, exemplarily showing the measured application-level latency for the vehicle registration uplink signal. The blue line represents the latency for each message sent from the vehicle to the VCoC. The red line is the average latency that is slightly below 100 ms. This plot shows that there is a considerable network jitter can be observed, which is in a comparable order of magnitude to the changes in transfer rates of video data. From these graphs, it follows that data will arrive late at the VCoC, which may negatively impact the ToD task performance of the operator. It is expected that these changes in data rates and latency are less severe with a 5G connection.

### ACCA

**Fig. 8:**
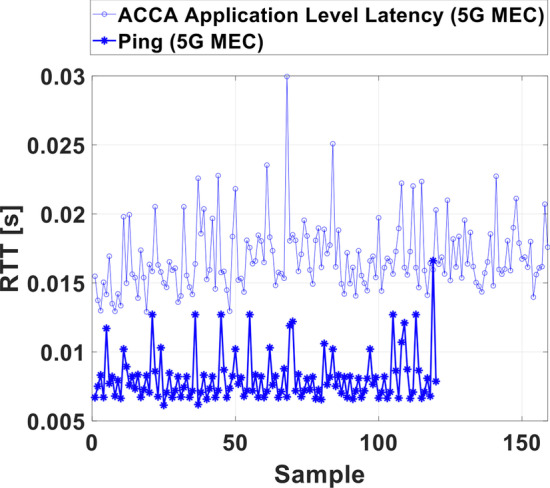
Ping RTT and ACCA application-level latency

Figure 8 shows consecutive measurement samples of network round trip times (RTTs) measured with the Ping tool and application-level latencies. The backend part of the use case was deployed on a MEC server located in Luxembourg, directly collocated with the core network. Ping and application-level latency measurements were executed right after another, not at the same time while the vehicle was not moving.

Ping RTTs are between 6.7 and 8.3 ms with some outliers around 12–13 ms and one outlier at 16.7 ms. The mean value is 8.1 ms. The variance between 6.7 and 8.3 ms is in the range of the time division duplex (TDD) frame requiring 2.5 ms for a full uplink/downlink period. Downlink period duration is about 2 ms and uplink period duration is about 0.5 ms long. An uplink packet therefore experiences 2 ms worst-case delay when waiting for the downlink direction to end while a downlink packet needs to wait up to 0.5 ms. Further Ping RTT delays result from fronthaul delays from the cell sites to the Core network in Luxembourg City (~ 30 km), from processing and transmission in the Core network and from the Ping application and the system hosting it.

The application-level latency is 16.8 ms on average with 0.3 ms 95% confidence interval obtained from separating the 157 measured samples into five batches. The additional delay of 8.7 ms likely originates from processing delays in the applications on the vehicle and the MEC-hosted backend.

Further trials will provide more insights on how different components of the end-to-end communication path contributes to the overall application-level latency. Results for moving vehicles were not presented but can be found in [22]. The path that was chosen was not appropriate due to very low radio signal quality as the distance to the serving cell got too large before reaching a motorway exit allowing to turn around. The trials are therefore being repeated on a rural road with two cells belonging to different MNO networks. This way, also seamless handover cross-border/-MNO handover between the two networks will be evaluated.

## Summary

This paper has summarized the main goals and activities conducted in 5GCroCo, just before the initiation of trials. 5GCroCo is an Innovation Action, partially funded by the European Commission, where key European partners from both the telco and automotive industries join efforts to trial and validate 5G technologies at large scale in a cross-border setting with the mission to reduce uncertainties before CCAM services running on top of 5G communication infrastructures can be offered to the market.

The trials focus on validating 5G key features in three different use cases: (1) tele-operated driving, (2) high-definition maps for autonomous driving and (3) anticipated cooperative collision avoidance. The main KPIs for these uses cases have been introduced in this paper, and the validation along the trials focuses on assessing the suitability of 5G to meet those requirements. In addition to the technical validation of 5G, 5GCroCo also aims at identifying business opportunities and defining new business models for disruptive CCAM services which can be possible thanks to 5G technology, as well as ensuring the appropriate impact into relevant standardization bodies both from the telco and automotive sectors.

This paper has also provided a first set of preliminary results for both ToD and ACCA use cases. While still not conclusive, the first round of test and trials have turned very useful to obtain important lessons to execute the second round of trials with higher accuracy in 2021, as it has been discussed and presented in this paper.

## Data Availability

Data sharing not applicable to this article as no datasets were generated or analysed during the current study.

## Abbreviations

3GPP: 3rd Generation Partnership Project
4G: Fourth-generation
5G: Fifth-generation
5GAA: 5G automotive association
5GCroCo: 5G cross-border control
5G NR: 5G new radio
5G-PPP: 5G public private partnership
ACCA: Anticipated cooperative collision avoidance
CCAM: Cooperative connected and automated mobility
C-ITS: Cooperative intelligent transport systems
E2E: End-to-end
EPC: Evolved packet core
EPS: Evolved packet system
EC: European Commission
eNB: Evolved node B
ETSI: European Telecommunications Standards Institute
gNB: Next-generation node B
GPS: Global positioning system
HD: High definition
ISO: International Organization for Standardization
IT: Information Technology
ITS: Intelligent transport systems
KPI: Key performance indicator
LTE: Long-term evolution
MEC: Mobile edge computing
MNO: Mobile network operator
OEM: Original equipment manufacturer
P-GW: Packet data network gateway
QoS: Quality of service
RAN: Radio access network
RAT: Radio access technologies
SDN: Software-defined network
S-GW: Serving gateway
ToD: Tele-operated driving
UC: Use case
V2X: Vehicle to everything
